# Meta-Ribosomalomics: RNA Sequencing Is an Unbiased Method for Parasite Detection of Different Sample Types

**DOI:** 10.3389/fmicb.2021.614553

**Published:** 2021-06-21

**Authors:** Claudia Wylezich, Dirk Höper

**Affiliations:** Friedrich-Loeffler-Institut, Institute of Diagnostic Virology, Greifswald-Insel Riems, Germany

**Keywords:** eukaryotic and prokaryotic diversity, parasites, ribosomal RNA, shotgun metagenomics, subtyping, taxonomic classification

## Abstract

In this perspective article, we review the past use of ribosomal sequences to address scientific and diagnostic questions. We highlight a variety of sequencing approaches including metagenomics and DNA barcoding and their different demands and requirements. Meta-ribosomalomics is introduced as an unbiased approach to exploit high-throughput sequencing datasets for eukaryotic and prokaryotic ribosomal sequences. Prerequisites, benefits, drawbacks, and future perspectives are elaborated and compared to other sequencing approaches.

## Introduction

Sequencing of isolates and complex samples using extracted DNA has become a standard technique in microbiology and diagnostic laboratories. A major breakthrough in microbiology enabled the culture-independent characterization of microorganisms (i.e., single-celled eukaryotes and prokaryotes). An abundance of gene sequences was generated for either diversity surveys or diagnostic purposes. In microbial ecology, a PCR-based investigation of environmental samples was established, subsequently called meta-barcoding (amplicon-sequencing of short marker sequences). However, the application of PCR with the necessity of oligonucleotide primers introduces biases in the results of such analyses. The untargeted investigation of genomes of complex samples was alleviated by the introduction of new sequencing technologies and high-throughput sequencing (HTS). This approach, also known as metagenomics, can likewise be performed with extracted DNA (DNA-sequencing). Extracted RNA and subsequent depletion of ribosomal RNA (rRNA) is typically used to investigate the transcriptome of an organism or a complex sample, termed transcriptomics or meta-transcriptomics, respectively. In contrast, metagenomic analyses can also be performed on any RNA sample without depletion of the rRNA; in this case, the occurring ribosomal nucleic acids lead to sequencing datasets including reads of prokaryotic and eukaryotic rRNA. These reads comprise sequences of all rRNA molecules, i.e., the small subunit (SSU; comprising the historically defined 16S and 18S molecules) and large subunit (LSU; historically 23S, 28S) as well as internal transcribed spacer regions and 5S rRNA. As the majority of such RNA-based metagenomics datasets consists of ribosomal RNA, sequencing of the ribosomalome (all rRNA molecules of a complex sample) is dubbed meta-ribosomalomics. From our perspective, this approach has many benefits and advantages over primer-dependent meta-barcoding and DNA-based metagenomics. As we present and discuss here, it can be an unbiased alternative to address diagnostic and ecological issues while allowing the parallel investigation of prokaryotic and eukaryotic diversity and the mutual interdependencies between both groups.

## Sequencing of Ribosomal RNA—From Past to Future

**FIGURE 1:**
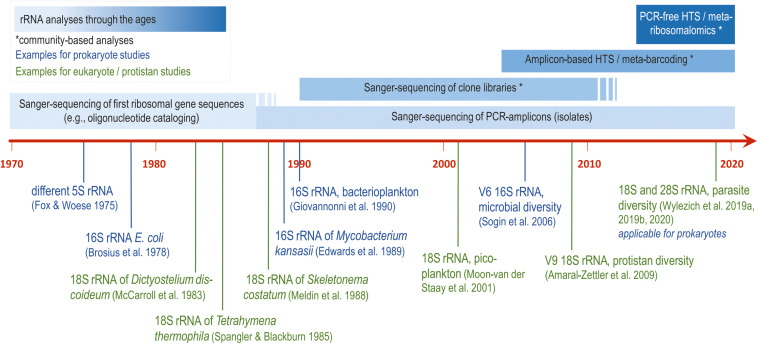
Milestones of generating small-subunit ribosomal RNA gene sequences of prokaryotes and protistan eukaryotes to address phylogenetic and ecological questions (with references for the first application each; Fox and Woese, 1975; Brosius et al., 1978; McCarroll et al., 1983; Spangler and Blackburn, 1985; Medlin et al., 1988; Edwards et al., 1989; Giovannoni et al., 1990; Moon-van der Staay et al., 2001; Sogin et al., 2006; Amaral-Zettler et al., 2009; Wylezich et al., 2019a, b, 2020).

Sequencing of ribosomal RNA (rRNA) genes for phylogenetic purposes was in the focus for almost half a century. The small subunit (SSU) of rRNA genes of prokaryotes (16S rRNA gene) were intensely studied in the nineteen-seventies using oligonucleotide catalogs and first available sequencing methods (reviewed by Woese, 1987). About one decade later, small subunit of rRNA genes of single-celled eukaryotes (18S rRNA gene) were studied for the first time (see Figure 1 for details).

The development of the enzymatic polymerase chain reaction (PCR) in 1987 (Mullis and Faloona, 1987) revolutionized the repertoire of molecular methods and molecular diagnostics, and was also the starting point for the road to success for the characterization of ribosomal sequences and their use in molecular phylogeny. With the introduction of PCRs of complex environmental samples and their subsequent separation and amplification via bacterial cloning (clone libraries), plenty of complete and nearly complete SSU rRNA gene sequences became available and enabled addressing ecological research questions using the taxa composition derived from the sequence composition. The advent of new sequencing technologies and the application of HTS allowed massive parallel sequencing of environmental sequences circumventing their separation via bacterial cloning. However, in contrast to sequencing of isolates and environmental clones, only short fragments (variable regions, e.g., prokaryotic V3 or V6 and eukaryotic V4 or V9 SSU rDNA; Figure 1) instead of nearly complete genes were sequenced. Conversely, the usage of short sequences for phylogenetic reconstructions may be disadvantageous regarding the phylogenetic resolution of taxa (e.g., Choi and Park, 2020). A general drawback of the application of PCR for the analysis of prokaryotic and eukaryotic community composition is the primer bias (e.g., Geisen et al., 2015; Clarke et al., 2017 and references therein). This PCR bias in the analysis of environmental systems is based in part on incomplete sequencing of the rRNA molecules and in part on the inability of the universal primers to capture and hence amplify certain specific or lesser abundant molecules. Taken together, even potentially important taxa maybe discriminated by the use of universal primers. In addition, read numbers in sequencing datasets do not represent the initial cell abundance of a sample, as revealed by the inclusion of mock communities (Geisen et al., 2015; Wylezich et al., 2018a). This impedes the quantitative analysis of read numbers. Hence, the molecularly described diversity of a habitat depends on the choice of primers and gene region (Choi and Park, 2020) and is mostly a fragmentary picture. This distorts comparisons of results obtained from different studies using different primers.

Nevertheless, ribosomal sequences are the most used molecular markers to date. A search for prokaryotic and eukaryotic SSU rRNA gene sequences in GenBank (search criteria: ((((((rRNA) OR ribosomal RNA) AND 18S) OR 16S) OR SSU) NOT genome) NOT probe) on September 29, 2020, resulted in 9,269,968 entries. This is much more than for other often-used barcoding markers, for example cytochrome c (1,569,826 entries; search criteria: ((cytochrome c) NOT genome) NOT probe), or cytochrome oxidase 1 (2,799,587 entries; search criteria: (cytochrome oxidase 1) NOT genome) NOT probe). The above-mentioned number of SSU rRNA gene sequences includes many complete or nearly complete sequences. In addition to sequences in GenBank, plenty of short ribosomal sequence fragments of the variable barcoding regions (e.g., eukaryotic V4 and V9, prokaryotic V6) are deposited in short read archives (SRA^1^) and even boost the number of available SSU rRNA reference sequences.

## Case Studies of Meta-Ribosomalomics for Parasite Detection

Diagnostic assays for the detection of pathogens including parasites typically target antigen (serologic detection and characterization) or nucleic acids (genetic detection and characterization); however, both approaches often lack standardization (Murray and Cappello, 2008). This is especially true for PCR assays for which different primer systems exist. Ribosomal sequences are used in clinical diagnostics from the early beginning and the reference database for ribosomal sequences of bacteria and parasites is huge. Meta-ribosomalomics can therefore be useful for parasite detection.

To overcome the limitations of the used primers, meta-ribosomalomics approaches hold a great promise. In a proof-of-concept study, we re-analyzed metagenomics datasets of fecal samples of virus-infected pigs derived from total RNA and successfully extracted complete 18S rRNA sequences of parasites (Wylezich et al., 2019a). Since the approach is PCR-free and does not rely on specific primers, it was possible to synchronously detect different parasites in one sample. Importantly, with this approach we could separate *Blastocystis* subtypes from each other and reported subtype 15 for the first time in pig feces. Hence, a hypothesis-free approach can be important to uncover the full diversity of species present in a sample which could be overlooked in case an expected species was detected and the analysis for other species stopped thereafter. In a follow-up study, the method was applied to fecal and organ samples pre-diagnosed for parasites (Wylezich et al., 2020). Diagnoses could be confirmed in nearly all cases. Further taxa (*Dientamoeba, Iodamoeba, Endolimax, Hymenolepis*) were synchronously recorded in addition to the pre-diagnosed ones. These results show the broad applicability of the approach and its discriminative power, which of course depends on the specificity of the *in silico* sequence analyses and the available reference sequences. Noteworthy mentioning that the sensitivity also depends on the accessibility of the nucleic acids of the different pathogens, as stressed by Wylezich et al. (2018b).

Beside gut samples, organ samples of mice were screened for parasites using meta-ribosomalomics. The 18S rRNA extracted from the resulting dataset allowed the molecular characterization of *Klossiella* cf. *muris* (available under accession numbers MT664760 and MT664769) using a complete 18S rRNA gene sequence for the first time. It exhibits a sequence identity of only 91.3% to the partial 18S rRNA gene of *K. equi* (unpublished results).

Furthermore, we characterized a parasite in Malpighian tubules samples of bees via meta-ribosomalomics. This allowed us for the first time the phylogenetic characterization of the bee parasite *Malpighamoeba mellificae* based on 18S rRNA retrieved from a complex sequencing dataset (Wylezich et al., 2019b). The actin gene could also be extracted from the dataset and supports the 18S rRNA phylogeny of *M. mellificae* as sister taxon of *Micriamoeba tesseris* within the class Tubulinea (Amoebozoa). Using 18S rRNA, a diagnostic PCR assay could be established for the first time for this often neglected bee parasite (unpublished results).

## Retrieving Ribosomal RNA Sequences From Field Samples

Meta-ribosomalomics is also applicable for field samples, for which typically amplicon-based 18S rRNA genes are used (e.g., Choi and Park, 2020). In proof-of-concept studies, we tested tap water samples (Wylezich et al., 2018b, and unpublished results) and groundwater samples (unpublished results). The datasets are currently being analyzed. First results indicate a high yield of 16S and 18S rRNA sequences that can be used to draw phylogenetic reconstructions and ecological considerations.

DNA-based metagenomics was used to investigate archaeal communities in a river estuary (Zou et al., 2020). Several genomes of ammonia-oxidizing Archaea were assembled from shotgun metagenomics datasets. 16S and 23S rDNA gene sequences retrieved from these assembled genomes, instead via PCR and amplicon-sequencing, were subsequently used for a phylogenetic characterization of the taxon.

In a direct comparison of SSU rDNA data from PCR-generated metabarcoding with sequence fragments extracted from DNA-based shotgun metagenomics sequencing datasets, the latter provided more realistic estimates in terms of microbe community diversity and allowed a better representation of the rare biosphere taxa (Logares et al., 2014). This study points to the fact that a PCR step clearly biases the outcome of community analyses and can outcompete low-abundance taxa.

## Discussion

Easy to amplify ribosomal RNA genes (rDNA) are well known and have a long tradition as genetic markers for phylogenetic assignments. The ease of use gave rise to huge reference databases for ribosomal genes (e.g., SILVA ribosomal RNA database, Quast et al., 2013), accompanied by the development of numerous algorithms and software for the analysis of rRNA sequence data, for instance RNAmmer (Lagesen et al., 2007), Barrnap^2^, SortMeRNA (Kopylova et al., 2012) or PhyloFlash (Gruber-Vodicka et al., 2020). Ribosomal RNA (rRNA) is an abundant biomarker that can be used in clinical diagnostics in order to improve the detection limit of PCR-diagnostics in contrast to the use of rDNA (Hanron et al., 2017). This is due to the enormous abundance of rRNA transcripts in RNA-based datasets in contrast to rDNA in DNA-based datasets (Wylezich et al., 2019a and references therein). The meta-ribosomalomics approach takes advantage of the large amount of rRNA sequences in RNA-based datasets. Another advantage (see Table 1) is the complete absence of any primer bias since no target specific primers are applied to amplify target genes. Untargeted (PCR- and primer-free) approaches are well suited to catch hitherto unknown, unrelated or unexpected sequence types (Wylezich et al., 2019a, b, 2020).

**TABLE 1:** Overview of the prerequisites, current benefits and drawbacks of the meta-ribosomalomics approach.

**Prerequisites**
• RNA-friendly laboratory conditions are important to avoid RNA degradation during sample processing • Establishment and validation of the approach per laboratory • Bioinformatics analysis needs an additional assembly step (in contrast to meta-barcoding)
**Benefits**
• Applicable for parallel detection of prokaryotes and eukaryotes in one sample • No prior sequence information is necessary since it is an untargeted approach • No primer bias exists since it is a PCR-free and primer-free approach • Detection of unrecognized, unrelated taxa and co-infections is possible • Resulting sequences can be a basis for designing better targeted approaches (e.g., for subsequent quantification purposes) • Resulting datasets can be re-analyzed for protein-coding genes when more reference genomes of parasites are available
**Drawbacks**
• False-positives might be detected based on mis-assignments due to incorrectly curated genomes or erroneously named ribosomal sequences (database contaminations) • High initial cost for establishment and validation of the approach per laboratory
**Future perspective**
• The possibility of quantification of detected taxa via found reads needs to be evaluated

In terms of abundance estimates and taxonomic classification, metagenomics whole-genome sequencing (based on DNA) shows a clearly better performance than meta-barcoding with rRNA amplicons as recently shown with mock communities (Logares et al., 2014; Khachatryan et al., 2020). However, the performance of RNA-based metagenomics (meta-ribosomalomics) regarding quantification purposes has yet to be investigated. Furthermore, the use of RNA for HTS has the advantage of drawing an immediate picture of the sample in contrast to DNA that can be derived from different sources including dead organisms or extracellular DNA (Pochon et al., 2017; Cristescu and Hebert, 2018).

In the above-mentioned examples, the generic meta-ribosomalomics approach was promising for diagnostics of typical but also neglected parasites and gut protists (e.g., Wylezich et al., 2019a, b, 2020). In addition, ribosomal gene sequences can be retrieved from DNA-based metagenomics datasets as shown for a novel species of ammonia-oxidizing Archaea (Zou et al., 2020). Regarding parasite characterization, missing well-curated reference genomes hamper correct read assignments and often deliver false-positive hits (e.g., Wylezich et al., 2019a). Therefore, ribosomal RNA gene sequences retrieved from metagenomics datasets (meta-ribosomalomics) might be more reliable to assign to the correct taxon than many protein-coding genes.

We mainly tested meta-ribosomalomics in the field of clinical diagnostics with a focus on eukaryotes. However, the attractiveness of the method is the parallel identification of prokaryotes and eukaryotes in a given sample via a single snapshot method. Therefore, the method is well suited to investigate gut microbiota with their prokaryotic and eukaryotic key players in one approach elucidating their complexity and interdependencies (Chabé et al., 2017). Thus, at least compositional trends of different gut microbiota can be explored using ribosomal genes. Based on such trends, candidate protein-coding genes can be subsequently retrieved from the datasets to prove and to deepen the initial findings. In addition, the method can provide the species and subtype inventory of microbiota based on ribosomal sequences together with information on viruses included in feces intended to fecal transplantations.

## Author Contributions

CW and DH conceived the study, wrote the manuscript, and approved it for publication. Both authors contributed to the article and approved the submitted version.

## Conflict of Interest

The authors declare that the research was conducted in the absence of any commercial or financial relationships that could be construed as a potential conflict of interest.

## Footnotes

1 https://www.ncbi.nlm.nih.gov/sra

2 https://github.com/tseemann/barrnap
